# LC–MS/MS analysis of carcinogenic tobacco-specific nitrosamines in *Spodoptera litura* using the QuEChERS method

**DOI:** 10.1038/s41598-023-37656-2

**Published:** 2023-07-27

**Authors:** Jabez Raju Battu, Somala Karthik, Gummudala Yashaswini, Himanshu Thakur, Alagesan Keerthana, M. P. Shireesh Kumar, Morthala Shankara Sai Reddy

**Affiliations:** 1 Department of Entomology, CSK Himachal Pradesh Agricultural University, Palampur, Himachal Pradesh India; 2 Department of Entomology, PG College of Agriculture, Dr. Rajendra Prasad Central Agricultural University, Pusa, Bihar India

**Keywords:** Biological techniques, Analytical biochemistry, Mass spectrometry

## Abstract

Nicotine is a highly addictive alkaloid and a neurostimulator found in tobacco that causes addiction in humans and makes tobacco a high-demand commercial product. It is popularly used for recreational purposes and is a harmful substance (Oral LD_50_ value for rat is 50 mg/kg) and causes addiction. The metabolites of nicotine such as the Tobacco-specific Nitrosamines (TSNAs) are hazardous substances whose metabolites are highly electrophilic and form DNA adducts, which will initiate the process of carcinogenesis. TSNAs are formed during curing, storage and fermentation due to the nitrosation of nicotine and other tobacco alkaloids. TSNAs are used as biomarkers for cancer risk assessment in humans exposed to tobacco and its products. To determine the occasional formation of TSNAs in tobacco-feeding insects, 5th instar larvae of *Spodoptera litura* and their faeces were analyzed for the presence of *N*′-nitrosonornicotine (NNN), 4-(methylnitrosamino)-1-(3-pyridyl)-1-butanone (NNK), and 4-(methylnitrosamino)-1-(3-pyridyl)-1-butanol (NNAL) along with the stored tobacco leaves (PT-76) using an Agilent 6470B LC–MS/MS system following ISO/DIS 19290:2015 protocol. The larvae are extracted in a buffered acetonitrile–water extraction and the amount of TSNAs are quantified in multiple reaction monitoring (MRM) mode. 20 $\upmu$l of each extracted and cleaned up sample was injected into the LC–MS/MS system for quantification. The Limit of Detection (LOD) and Limit of Quantification (LOQ) were 0.001 mg/kg and 0.005 mg/kg for all the tested nitrosamines. NNN was found to be 0.361 mg/kg, 0.340 mg/kg, and 5.66 mg/kg in insect whole-body samples, faeces, and tobacco leaves, respectively. NNK was found to be 0.060 mg/kg, 0.035 mg/kg and 0.93 mg/kg in insect whole body samples, faeces and tobacco leaves, respectively. However, NNAL was not detected in both the insect’s whole body and faeces. Recoveries ranged between 95 and 98% for all compounds when spiked at LOD and LOQ. The presence of TSNAs is a biomarker for cancer risk and their presence in insects would point to cancer risk assessment in tobacco feeding insects and any possible TSNA-detoxifying pathways in insects that might prevent mutagenesis caused these compounds.

Tobacco-specific Nitrosamines (TSNAs) are a group of carcinogens found in tobacco and tobacco products^1^. They are formed during tobacco curing, storage, and fermentation by nitrosation of tobacco alkaloids such as nicotine, nornicotine, anabasine, and anatabine. TSNAs are not present in fresh leaves of tobacco and their formation starts a few days after harvesting^2–4^. The TSNAs include 4-(methylnitrosamino)-1-(3-pyridyl)-1-butanone (NNK), *N’*-nitrosonornicotine (NNN), *N’*-nitrosoanabasine (NAB) and *N’*-nitrosoanatabine (NAT). The former two are potent carcinogens (Group 1- highly carcinogenic) and are believed to be associated with majority of the lung cancers in smokers^5,6^. Boyland et al.^7,8^ were the first to demonstrate the carcinogenic activity of NAB in rats and NNN in mice. NNN & NNK are reported to cause oesophageal tumors, tumors of the olfactory epithelium, tracheal tumors, lung adenomas and adenocarcinomas in different animals as well as humans^6,9^. 4-(methylnitrosamino)-1-(3-pyridyl)-1-butanol (NNAL) is a metabolite of NNK and is reported to cause lung cancers in humans. Patients having lung cancers had significantly higher levels of urinary NNAL^10^. Consuming tobacco in any form renders humans exposed to the risk of acquiring cancers.

Insects feeding on tobacco are not affected by the mutagenic compounds present in the tobacco leaves. Experiments on tobacco-feeding insects suggested that in a majority of insects either nicotine is not metabolized by the insects or nicotine and other likely harmful tobacco alkaloids are rapidly excreted by the insects before lethal concentrations are accumulated^11,12^. Investigations to determine the nicotine metabolism in the tobacco hornworm, *Manduca sexta* revealed that nicotine is metabolized into cotinine-N-oxide and is rapidly excreted along with the free nicotine that is not metabolized^13^. It is believed that midgut enzymes like the cytochrome P450 monooxygenases and Glutathione S-transferases are responsible for the rapid excretion of nicotine in *Helicoverpa assulta*^14,15^. The present investigation was aimed at the determination of TSNAs and their fate in a tobacco feeding insect, *Spodoptera litura* commonly referred to as tobacco cutworm. Tobacco leaves fermented in the gut of this insect should result in the formation of TSNAs as they are formed during the curing and fermentation of tobacco leaves. This is probably the first time TSNAs are being analyzed in insects.

Chromatographic techniques were used for separation and quantifying analytes from admixtures of compounds in a solution. But coupling mass spectrometry to the chromatographic techniques has made the analyzing procedure highly accurate. Mass spectrometry (MS) uses charge-to-mass (*m/z*) such that any fragment ions formed during the process of ionization would not go unaccounted for^16^. Especially, MS equipped with triple quadrupoles can be highly specific for detecting many targeted analytes. This is called multiple reaction monitoring (MRM), which we have used for analyzing TSNAs in *S. litura* larvae. Several researchers used this method to analyze nicotine and other tobacco-derived harmful compounds in biological matrices. Alasmari et al.^17^ used UPLC-MS/MS for quantifying nicotine and cotinine in mice serum. Metabolic pathways can also be elucidated using these chromatographic techniques when coupled with mass spectrometry. Yin et al.^18^ used ultrahigh-resolution matrix-assisted laser desorption/ionization (MALDI) to study the metabolites of the drug Simvastatin in rat body fluids. Amer et al.^19^ used LC–MS/MS method for quantifying a drug called Masitinib in rat liver microsomes matrix and rat urine. Metabolism of drugs and the pathways associated with their metabolism can be studied using LC–MS/MS^20–22^. The present study also uses LC–MS/MS for analyzing TSNAs in the tobacco feeding caterpillar, *S. litura*.

## Materials and methods

### Reagents and chemicals

Methanol, Ammonium acetate (E. Merck, India Ltd.), Agilent QuEChERS extraction kit (p/n 5982-5755CH) and dispersive SPE kit (p/n 5982–5022) was used for extracting the samples and the internal standards of NNN and NNK procured were mentioned below in the Table 1. Milli-Q water was used for the preparation of the samples.

**Table 1:** Internal and reference standards of TSNAs.

S.No	Chemical	CAS Number	Company	Purity (%)
1	( ±)-N′-Nitrosonornicotine (NNN) solution	80508-23-2	Chem Service Inc	99.5
2	4-(methylnitrosamino)-1-(3-pyridyl)-1-butanone (NNK) solution	64091-91-4	Chem Service Inc	98.2
3	3 4-(methylnitrosamino)-1-(3-pyridyl)-1-butanol (NNAL) solution	76014-81-8	Chem Service Inc	99.5
4	Quinoline-d7	91-22-5	Sigma Aldrich	98

### Sample preparation

Tobacco seeds (PT 76) were procured from the local market, Samastipur and sown in pots. *S. litura* larva were collected from the tobacco fields and reared on tobacco leaves (PT 76) for five generations under controlled conditions (25 ± 1 °C; 70% RH). All life stages of five generations were found to be healthy and free of malformations. 5th instar larvae of *S. litura* from the 5th generation and their faeces were collected and stored at −20 °C in a freezer. Leaves of the tobacco were collected and stored at −20 °C in a freezer for 3 days to allow the formation of TSNAs.

#### Insect whole body and faeces

The extraction method was followed as given by Saremba et al.^23^, which was adapted from a QuEChERS method (AOAC 2007.01, Agilent Inc. ®) as given by Chang^24^ for extracting nicotine and its metabolites in *Trichoplusia ni.* Tissues and faeces of five larvae were used for sample preparation. Insect whole body specimens and faeces (1 g representative sample was made from 5 larvae) were homogenized and extracted in a buffered acetonitrile–water (0.5 mL:0.5 mL) extraction and this extract is added to the Agilent AOAC extraction kit and is vortexed for 5 s (pH was adjusted to 11 by using NaOH) and centrifuged or 5 min at 5000 rpm. The supernatant from the extracts was collected and vortexed on a dSPE cleanup column (AOAC 2007.01) to remove pigments, lipids, and proteins then centrifuged for 5 min at 10,000 rpm. The top supernatant layer was collected and filtered through 0.2 µm PTFE syringe filter to remove any particulates and 20 µL was injected into autosampler vial.

#### Tobacco leaves

Tobacco leaves were extracted following the protocol used by Li et al.^25^ with slight modifications. 250 mg leaf sample was added with 50 $\upmu$l internal standard solution, homogenized and filtered through a 425 $\upmu$m sieve and extracted in ammonium acetate using the QuEChERS method (AOAC 2007.01, Agilent Inc. ®) and centrifuged at 5000 rpm for 5 min. The extractant is directly filtered into the injection vials using a 0.2 $\upmu$m PTFE syringe filter.

### Preparation of standard solutions

1 mg/ml standard solutions were used to prepare stock solutions (1 ng/ml to 20 ng/ml) in LC–MS grade methanol through serial dilutions and were stored at −4 °C until further usage. The calibration curves of NNN, NNK, and NNAL are given in Supplementary 1.

### Instrumentation

The LC–MS/MS system (Agilent 6470B TQ LC/MS) consisted of a UHPLC (Agilent 1290 Infinity II) and an atmospheric pressure ionization triple quadrupole mass spectrometer (Agilent 6470 LC/TQ) equipped with electron spray ionization (ESI) Agilent Jetstream source. The UHPLC has a Zorbax Eclipse Plus-C18 column (3 mm inner diameter × 100 mm long × 1.8 $\upmu$m pore size). For quantification of TSNAs, LC–MS/MS was run with mobile phase A (water: 0.1% formic acid (2:98 v/v) with pH = 3) and mobile phase B (100% methanol: 0.1% formic acid with pH = 3) at a flow rate of 0.5 ml/min (isocratic flow) coupled to a triple quadrupole Mass spectrometer with a capillary voltage of 4000 V, Nozzle voltage of 500 V, nebulizer gas nitrogen with a pressure of 45 psi, the gas temperature of 300 °C and sheath gas temperature of 350 °C and gas flow and sheath gas flow of 8L/min and 11L/min, respectively. It was operated in electrospray positive mode and data collection was done in MRM mode. The LC–MS/MS system had an injection volume of 20 $\upmu$l and the total run time was 20 min. The column temperature was maintained at 40 °C. ISO/DIS 19,290:2015 method was used for the analysis of TSNAs.

### Data analysis

Data analysis was done using Lab Solutions software (Shimadzu).

**Ethical statement: **This article does not contain any studies involving humans/animals/plants that need approval from ethical committee. The plant material may be made available on request. The plant material used in this study complies with the guidelines of IUCN Policy Statement on Research Involving Species at Risk of Extinction and Convention on the Trade in Endangered Species of Wild Fauna and Flora. No plant material is not obtained from endangered species.

## Results and discussion

### Method validation

The linearity was evaluated by preparing a standard solution with different concentrations with different mobile phases and injecting them in the same operating procedure. The calibration curves of NNN, NNK and NNAL were found to have a linear relationship between (y) and (x) which is denoted in Supplementary 1. The precision was evaluated by reproducibility and repeatability of the method which is represented by relative standard deviation (RSD). For quality control, NNN, NNK and NNAL solutions of 0.1 ppm concentration are injected into the LC–MS/MS system along with the blank samples. The Accuracy was over 100% and the precision is less than 5% (Supplementary 2)**.**

**Figure 1:**
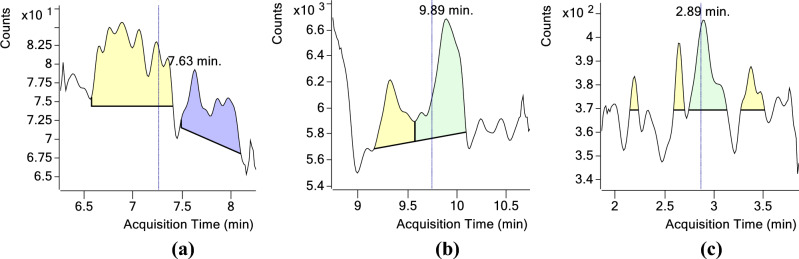
Chromatogram of blank samples (**a**) NNN, (**b**) NNK and (**c**) NNAL.

**Figure 2:**
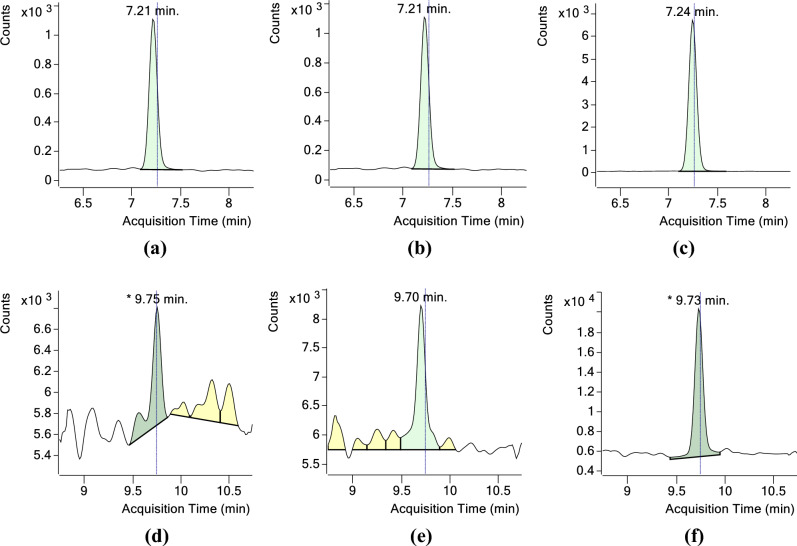
Chromatograms of NNN (103.1 −  > 75.1) and NNK (131.0 −  > 43.1) in insect and tobacco samples (**a**) NNN—*S. litura* whole body, (**b**) NNN—*S. litura* faeces (**c**) NNN—tobacco leaves (**d**) NNK—*S. litura* whole body, (**e**) NNK—*S. litura* faeces (**f**) NNK—tobacco leaves.

The reference standards are spiked at 0.001 and 0.005 mg/kg and recoveries obtained were 95–98% for all the three TSNAs being tested. When spiked at the concentrations of 0.01–0.2 $\upmu$g/ml (ppm), the mean recoveries ranged from 95.49 to 106.70%, 93.20 to 109.73% and 98.19 to 101.74% for NNN, NNK and NNAL, respectively (Supplementary 3). The limit of detection and limit of quantification were worked out to be 0.001 and 0.005 mg/kg, respectively for all the TSNAs viz. NNN, NNK and NNAL. The RSD% was less than 2% for all the internal standards tested. The equations showing linearity of the standards is given in Table 2. The retention time of the TSNAs and their levels in insect whole body and faeces are given below in Table 3. The chromatogram of the samples analyzed is also given below in Figs. 1 and 2.

**Table 2:** Linearity of NNN, NNK and NNAL.

Standard	R^2^	Calibration curve	LOD (mg/kg)	LOQ (mg/kg)
NNN	0.9954	276.78X − 260.03	0.001	0.005
NNK	0.9955	658.33X + 2257.95	0.001	0.005
NNAL	0.9990	823.82X + 510.48	0.001	0.005

**Table 3:** Retention time and concentration of NNN and NNK in the analyzed samples (average of three replicates).

S.No	Sample	Analyte	Retention time (min)	Quantification (mg/kg)	RSD%
1	*S. litura* whole body	NNN	7.23	0.361 ± 0.007	1.93
		NNK	9.75	0.060 ± 0.001	1.67
		NNAL	–	Not Detected	–
2	*S. litura* faeces	NNN	7.21	0.340 ± 0.006	1.76
		NNK	9.70	0.035 ± 0.0004	1.14
		NNAL	–	Not Detected	–
3	Tobacco leaves (PT 76)	NNN	7.24	5.659 ± 0.100	1.77
		NNK	9.73	0.933 ± 0.018	1.92

Internal standard (Quinoline-d7) was spiked in 6 replicates at LOQ i.e., 0.01 ppm and recoveries obtained were similar to those of the compounds of interest. Matrix effects observed as either ion suppression or ion enhancement occur usually because of the presence of co-eluting compounds present in the same matrix^26^. Matrix effects on TSNAs quantification was evaluated using internal standard solution through post column infusion method and was found to be 0%. Further, known concentration of internal standard solutions were also added to the extracted samples and analyzed for the TSNAs and the final concentration showed that there are no significant matrix effects on TSNA quantification.

$${\text{Matrix effect }} = {\text{ A}} - {\text{B}}/{\text{A }} \times { 1}00$$

where A is the peak area of an analyte in a standard solution and B is the is the peak area of the analyte in a sample (Blank spiked with analyte in the same concentration as the standard solution).

Uncertainty studies (supplementary material 6) were performed on the analytes using the procedure as described by Klu et al.^27^ and the uncertainties measured for reference standards is given in Table 4. The combined uncertainty was less than 7% for all the analytes. At 95% level of confidence, the expanded uncertainty for NNK, NNN and NNAL is 0.667, 0.631 and 0.639 $\upmu$g/kg, respectively.

**Table 4:** Uncertainty measurement.

S. No	Compound name	Rang of testing	Uncertainty measurement
1	NNAL	10 to 150 $\upmu$g/kg	± 0.63 $\upmu$g/kg
2	NNN	10 to 150 $\upmu$g/kg	± 0.62 $\upmu$g/kg
3	NNK	10 to 150 $\upmu$g/kg	± 0.66 $\upmu$g/kg

### TSNAs in insect samples

Quantification of TSNAs was done in multiple reactions monitoring (MR mode. The proposed fragmentation of parental ions is given in supplementary material 7. NNN was found to be 0.360 ± 0.000631, 0.340 ± 0.000631 and 5.66 ± 0.000631 mg/kg in insect whole body, faeces and tobacco leaves, respectively. NNK was observed to be 0.060 ± 0.000667, 0.035 ± 0.000667 and 0.093 ± 0.000667 mg/kg in insect whole body, faeces and tobacco leaves, respectively. NNAL was below quantifiable limits in all the three specimens.

NNN and NNK are detected in both insect whole body and faeces. The data indicates the rapid excretion of the carcinogenic compounds just as soon as they are formed in a similar fashion to the rapid excretion of nicotine and its metabolite cotinine-N-oxide as reported by Snyder et al.^13^. NNAL, a metabolite of NNK is not detected in both the insect’s whole body and faeces indicating that NNK is not metabolized in *S. litura*, similar to the analysis of nicotine metabolism in cigarette beetle, *Lasioderma serricorne* conducted by Farnham et al.^12^ which revealed that nicotine is neither sequestered nor detoxified but is rather excreted. Pérez-Ortuño et al.^28^ reported that the mean NNN and NNK concentration in the oral fluid of people who smoked at least 1 cigarette per day was 118 and 6.6 pg/ml. Kavvadias et al.^29^ reported NNN mean concentration in the urine of smokers as 7.2 pg/ml. The NNN concentration we detected in *S. litura* larvae is 0.360 mg/kg in the whole body and 0.340 mg/kg in insect faeces which is approximately 4778 times more than what was reported in human urine by Kavvadias et al.^29^. The presence of TSNAs in the human urine or oral fluid is considered a biomarker for cancer risk^28^.

The probability of insect getting cancer is very low owing to several factors. First and foremost, the life span of an average insect is rather short for any cancer to be developed at all, secondly, insects are popularly known for their xenobiotic detoxification potential and any carcinogenic agent (like pesticides for example) could be detoxified. Moreover, the genome of the insect is of small size and therefore the chances for mutations are very less. Furthermore, insects undergo programmed cell death throughout their life cycle whenever they undergo metamorphosis which will likely prevent any tumors^30^. But again, insects can get cancer as well. Peto’s paradox states that there is no correlation between the body size and cancer risk^31^, but we are inconclusive as of now to use this to answer whether insects get cancer . There had been reports of tumors in insects which likely explain this. Harker^32^ reported excessive endocrine secretions from sub-oesophageal ganglion-induced tumors in the midgut of *Periplaneta americana*. Federley^33^ reported male-killing in the species hybrids of the butterfly *Pygaera pigra* (Notodontitade: Lepidoptera). Male larvae of the species hybrids are killed due to cancer before they even reach pupation. TSNAs are potent carcinogens and reported to be associated with lung cancers in smokers^5,6^. To determine whether TSNAs possess a significant cancer risk in insects, deeper investigation is required. For example, insect cell lines such as the Sf9 cell line can be used to evaluate the mutagenicity of these compounds and their metabolites on insect cells.

## Conclusion

Tobacco-specific Nitrosamines are carcinogenic substances and are associated with various types of cancers. They are formed during the process of curing, storage and fermentation. TSNAs can also form in the midgut of tobacco-feeding insects, wherein ingested tobacco leaves are fermented. We have detected the presence of NNN and NNK in the insect’s whole body and faeces indicating their formation in tobacco-feeding insects. The genotoxic effects of TSNAs are widely reported in higher animals but there is no study conducted to investigate the effects of TSNAs on lower animals like invertebrates. This study prompts future investigations into how TSNAs are formed, metabolized, and detoxified (if any) in insects. This experiment should be conducted on all the tobacco-feeding insects to see how different insects have developed strategies to mitigate the genotoxic effects of TSNAs on them. In order to explore the impact of dietary risk linked with their feeding habits and the evolution of powerful xenobiotic detoxifying mechanisms in them as contrasted to that of the higher animals, it would be useful to utilize insects that feed on plants like tobacco that contain dangerous compounds.

## Supplementary Information

Supplementary Information.

## Data Availability

The datasets generated during and/or analysed during this study are included in this published article (and its Supplementary Information files).
