# Supplementary material for: LC–MS/MS analysis of carcinogenic tobacco-specific nitrosamines in Spodoptera litura using the QuEChERS method
Source: Sci Rep. 2023 Jul 27;13:12151. doi: 10.1038/s41598-023-37656-2 (PMC10374897; doi:10.1038/s41598-023-37656-2)
Supplement: Supplementary file 1 — Supplementary Information. [file 41598_2023_37656_MOESM1_ESM.doc]

|  |  |
| --- | --- |
| **(A)** |  |
| **(B)**    **(C)**  **Supplementary material 1: Calibration curves of a) NNK, b) NNN and c) NNAL**   | **Compound** | **Spiked concentration (ng/ml)** | **Mean ± SD**  **(n = 6)** | **Accuracy (%)** | **Precision (%)** | | --- | --- | --- | --- | --- | | NNN | 100 | 108.63 ± 0.34 | 108.63 | 0.31 | | NNK | 100 | 117.28 ± 0.49 | 117.28 | 0.42 | | NNAL | 100 | 98.46 ± 0.32 | 98.46 | 0.33 | | |

**Supplementary material 2: Precision and accuracy of LC-MS/MS method**

| **Compound** | **Spiked level (ppb)** | **Mean ± SD (n=6)** | **Recovery percentage** |
| --- | --- | --- | --- |
| NNN | 10 | 10.106 ± 0.196 | 101.06 |
| NNK | 10 | 9.466 ± 0.330 | 94.66 |
| NNAL | 10 | 10.85 ± 0.231 | 108.50 |

**Supplementary material 3: Recoveries of reference standards**

**(A) Chromatograms of NNN when spiked at 10, 25, 50, 100, 150 and 200 ppb concentrations (from left to right)**

**(B) Chromatograms of NNK when spiked at 10, 25, 50, 100, 150 and 200 ppb concentrations (from left to right)**

**(C) Chromatograms of NNAL when spiked at 10, 25, 50, 100, 150 and 200 ppb concentrations (from left to right)**

**Supplementary material 4: Chromatograms of analytical standards**

+ MRM (137.0 -> 81.1) QC_10.0 PPB.d S

…

Acquisition Time (min)

9

9.5

10

10.5

C

o

u

n

t

s

3

x10

3

4

5

6

7

8

9.71 min.

**Supplementary material 5: Chromatogram of stable isotope-labelled internal standard (Quinoline-d7)**

**Supplementary material 6: Quality control and uncertainty measurement of NNN, NNK &** NNAL spiked at 10 ppb

| **NNK** | | **NNN** | | **NNAL** | |
| --- | --- | --- | --- | --- | --- |
| **Replications** | **Recovery (ppb)** | **Replications** | **Recovery (ppb)** | **Replications** | **Recovery (ppb)** |
| **1** | 9.5215 | **1** | 10.3265 | **1** | 10.0214 |
| **2** | 9.0265 | **2** | 10.1155 | **2** | 10.2314 |
| **3** | 9.5542 | **3** | 9.9854 | **3** | 10.1125 |
| **4** | 9.3515 | **4** | 10.3254 | **4** | 10.0652 |
| **5** | 10.0215 | **5** | 9.8251 | **5** | 10.6321 |
| **6** | 9.3251 | **6** | 10.0587 | **6** | 10.0521 |
| **Average** | 9.4667 | **Average** | 10.1061 | **Average** | 10.1858 |
| **Standard Deviation** | 0.3303 | **Standard Deviation** | 0.1962 | **Standard Deviation** | 0.2307 |

**Supplementary material 6.1: Recoveries of analytical standards at 10 ppb**

| **Uncertainty** **measurement** **of** **NNAL** | |
| --- | --- |
| **Standard Uncertainty due to Standard Preparation (U_a_)** | 0.0942 |
| **Type B, U_b_** | |
| 1) U_b1_, Standard Uncertainty due to CRM | 0.000187 |
| 2) U_b2_, Standard Uncertainty due to Weighing Balance (10 mg) | 0.000431 |
| 3) U_b3_, Standard Uncertainty due to Volumetric Flask (10ml) | 0.004721 |
| 4) U_b4_, Standard Uncertainty due to Micro Pipette (1ml) | 0.045290 |
| 5) U_b5_, Standard Uncertainty due to Volumetric Flask (10ml) | 0.003462 |
| 6) U_b6_, Standard Uncertainty due to Micro Pipette (0.1ml) | 0.006289 |
| 7) U_b7_, Standard Uncertainty due to Volumetric Flask (10ml) | 0.009989 |
| **Standard Uncertainty due to Sample Preparation** | |
| 8) U_b8_, Standard Uncertainty due to Weighing Balance (2 gm) | 0.000096 |
| 9) U_b9_, Standard Uncertainty due to Micro Pipette (0.1ml) | 0.006000 |
| 10) U_b10_, Standard Uncertainty due to Measuring Cylinder (10 ml) | 0.085610 |
| 11) U_b11_, Standard Uncertainty due to Micro Pipette (1ml) | 0.053970 |
| **Standard Uncertainty due to Instruments** | |
| 12) U_b12_, Standard Uncertainty due to GC Oven (70-280°C) | 0.283412 |
| 13) U_b13_, Standard Uncertainty due to Syringe (10 µl) | 0.019624 |
| Combined uncertainty of Type B U_b_ =√U_b1_^2^+U_b2_^2^+U_b3_^2^+……….+U_b13_^2^ | 0.305304 |
| Combined Standard Uncertainty U_c_=√(Ua)^2^+(Ub)^2^ | 0.319500 |
| Expanded Uncertainty =Uc x k ( k = 2 at 95% confidence level) | 0.639001 |
| **UM at mean** | **0.639001** |

**Supplementary material 6.2: Uncertainty measurement of NNAL**

| **Uncertainty** **measurement** **of** **NNN** | |
| --- | --- |
| **Standard Uncertainty due to Standard Preparation (U_a_)** | 0.0801 |
| **Type B, U_b_** | |
| 1) U_b1_, Standard Uncertainty due to CRM | 0.000187 |
| 2) U_b2_, Standard Uncertainty due to Weighing Balance (10 mg) | 0.000431 |
| 3) U_b3_, Standard Uncertainty due to Volumetric Flask (10ml) | 0.004721 |
| 4) U_b4_, Standard Uncertainty due to Micro Pipette (1ml) | 0.045290 |
| 5) U_b5_, Standard Uncertainty due to Volumetric Flask (10ml) | 0.003462 |
| 6) U_b6_, Standard Uncertainty due to Micro Pipette (0.1ml) | 0.006289 |
| 7) U_b7_, Standard Uncertainty due to Volumetric Flask (10ml) | 0.009989 |
| **Standard Uncertainty due to Sample Preparation** | |
| 8) U_b8_, Standard Uncertainty due to Weighing Balance (2 gm) | 0.000096 |
| 9) U_b9_, Standard Uncertainty due to Micro Pipette (0.1ml) | 0.006000 |
| 10) U_b10_, Standard Uncertainty due to Measuring Cylinder (10 ml) | 0.085610 |
| 11) U_b11_, Standard Uncertainty due to Micro Pipette (1ml) | 0.053970 |
| **Standard Uncertainty due to Instruments** | |
| 12) U_b12_, Standard Uncertainty due to GC Oven (70-280°C) | 0.283412 |
| 13) U_b13_, Standard Uncertainty due to Syringe (10 µl) | 0.019624 |
| Combined uncertainty of Type B U_b_ =√U_b1_^2^+U_b2_^2^+U_b3_^2^+……….+U_b13_^2^ | 0.305304 |
| Combined Standard Uncertainty U_c_=√(Ua)^2^+(Ub)^2^ | 0.315638 |
| Expanded Uncertainty =Uc x k ( k = 2 at 95% confidence level) | 0.631276 |
| **UM at mean** | **0.631276** |

**Supplementary material 6.3: Uncertainty** **measurement** **of** **NNN**

| **Uncertainty** **measurement** **of** **NNK** | |
| --- | --- |
| **Standard Uncertainty due to Standard Preparation (U_a_)** | 0.1349 |
| **Type B, U_b_** | |
| 1) U_b1_, Standard Uncertainty due to CRM | 0.000187 |
| 2) U_b2_, Standard Uncertainty due to Weighing Balance (10 mg) | 0.000431 |
| 3) U_b3_, Standard Uncertainty due to Volumetric Flask (10ml) | 0.004721 |
| 4) U_b4_, Standard Uncertainty due to Micro Pipette (1ml) | 0.045290 |
| 5) U_b5_, Standard Uncertainty due to Volumetric Flask (10ml) | 0.003462 |
| 6) U_b6_, Standard Uncertainty due to Micro Pipette (0.1ml) | 0.006289 |
| 7) U_b7_, Standard Uncertainty due to Volumetric Flask (10ml) | 0.009989 |
| **Standard Uncertainty due to Sample Preparation** | |
| 8) U_b8_, Standard Uncertainty due to Weighing Balance (2 gm) | 0.000096 |
| 9) U_b9_, Standard Uncertainty due to Micro Pipette (0.1ml) | 0.006000 |
| 10) U_b10_, Standard Uncertainty due to Measuring Cylinder (10 ml) | 0.085610 |
| 11) U_b11_, Standard Uncertainty due to Micro Pipette (1ml) | 0.053970 |
| **Standard Uncertainty due to Instruments** | |
| 12) U_b12_, Standard Uncertainty due to GC Oven (70-280°C) | 0.283412 |
| 13) U_b13_, Standard Uncertainty due to Syringe (10 µl) | 0.019624 |
| Combined uncertainty of Type B U_b_ =√U_b1_^2^+U_b2_^2^+U_b3_^2^+……….+U_b13_^2^ | 0.305304 |
| Combined Standard Uncertainty U_c_=√(Ua)^2^+(Ub)^2^ | 0.333759 |
| Expanded Uncertainty =Uc x k ( k = 2 at 95% confidence level) | 0.667518 |
| **UM at mean** | **0.667518** |

**Supplementary material 6.4: Uncertainty studies of NNK**

| **Compound name** | **Precursor ion** | **Product ion** | **Dwell** | **Fragmentor (V)** | **Collision energy**  **(V)** | **Cell accelerator voltage**  **(V)** | **Polarity** |
| --- | --- | --- | --- | --- | --- | --- | --- |
| **NNAL** | **210.1** | **180.1** | **50** | **110** | **18** | **3** | **Positive** |
| **NNK** | **208** | **121.9** | **50** | **80** | **19** | **3** | **Positive** |
| **NNK** | **208** | **79** | **50** | **80** | **12** | **2** | **Positive** |
| **NNN** | **178** | **148** | **50** | **75** | **18** | **3** | **Positive** |
| **NNN** | **178** | **120** | **50** | **80** | **11** | **3** | **Positive** |
| **Quinoline-d7** | **137** | **81** | **50** | **110** | **35** | **3** | **Positive** |

**Supplementary material 7: Proposed fragmentation of parental ions**
